# Impaired Glucose Metabolism in Mice Lacking the *Tas1r3* Taste Receptor Gene

**DOI:** 10.1371/journal.pone.0130997

**Published:** 2015-06-24

**Authors:** Vladimir O. Murovets, Alexander A. Bachmanov, Vasiliy A. Zolotarev

**Affiliations:** 1 Department of physiology of digestion, Pavlov Institute of Physiology, Saint-Petersburg, Russia; 2 Monell Chemical Senses Center, Philadelphia, Pennsylvania, United States of America; Duke University, UNITED STATES

## Abstract

The G-protein-coupled sweet taste receptor dimer T1R2/T1R3 is expressed in taste bud cells in the oral cavity. In recent years, its involvement in membrane glucose sensing was discovered in endocrine cells regulating glucose homeostasis. We investigated importance of extraorally expressed T1R3 taste receptor protein in age-dependent control of blood glucose homeostasis *in vivo*, using nonfasted mice with a targeted mutation of the *Tas1r3* gene that encodes the T1R3 protein. Glucose and insulin tolerance tests, as well as behavioral tests measuring taste responses to sucrose solutions, were performed with C57BL/6ByJ (*Tas1r3*+/+) inbred mice bearing the wild-type allele and C57BL/6J-*Tas1r3^tm1Rfm^* mice lacking the entire *Tas1r3* coding region and devoid of the T1R3 protein (*Tas1r3-/-*). Compared with *Tas1r3*+/+ mice, *Tas1r3-/-* mice lacked attraction to sucrose in brief-access licking tests, had diminished taste preferences for sucrose solutions in the two-bottle tests, and had reduced insulin sensitivity and tolerance to glucose administered intraperitoneally or intragastrically, which suggests that these effects are due to absence of T1R3. Impairment of glucose clearance in *Tas1r3-/-* mice was exacerbated with age after intraperitoneal but not intragastric administration of glucose, pointing to a compensatory role of extraoral T1R3-dependent mechanisms in offsetting age-dependent decline in regulation of glucose homeostasis. Incretin effects were similar in *Tas1r3*+/+ and *Tas1r3*-/- mice, which suggests that control of blood glucose clearance is associated with effects of extraoral T1R3 in tissues other than the gastrointestinal tract. Collectively, the obtained data demonstrate that the T1R3 receptor protein plays an important role in control of glucose homeostasis not only by regulating sugar intake but also via its extraoral function, probably in the pancreas and brain.

## Introduction

The search for key regulators of blood or tissue glucose levels is relevant to treatments of diabetes, obesity, and metabolic syndrome. Sensing of glucose in viscera and brain is crucial for control of energy homeostasis. Cells involved in regulation of blood glucose concentration (the insulin-secreting β-cells of the pancreas, enteroendocrine L-cells of the small intestine, and glucose-excited neurons of ventromedial hypothalamus) share a metabolic mechanism of glucose sensing controlled by glucose transporters and glucokinase (hexokinase IV). This mechanism involves an increase of cytoplasmic glucose resulting in a series of intracellular events leading to a rise in the cytosolic ATP/ADP ratio and subsequent closure of ATP-sensitive potassium (K_ATP_) channels, which leads to cell depolarization [1, 2]. However, several lines of evidence, including pharmacological blockage of glucokinase and gene knockout of the Kir6.2 subunit of the K_ATP_ channel, strongly suggest that glucosensing involves additional signaling pathways that do not require intracellular metabolic processing of glucose, that is, K_ATP_-independent pathways [3–6]. In recent studies, membrane glucose-sensing mechanisms involving the T1R2/T1R3 heterodimeric complex of G-protein-coupled sweet taste receptor proteins [7, 8] and related intracellular transduction components, operating independently of cellular glucose transport and metabolism, were found in gastrointestinal, nervous, and endocrine cells regulating glucose homeostasis [9–17].

Results of *in vitro* and some *in vivo* studies confirm the role of T1R-related mechanisms in regulation of glucose metabolism. In cultures of enteroendocrine cells, these mechanisms involve insulinotropic hormones, or incretins: glucagon-like peptide 1 (GLP-1) and glucose-dependent insulinotropic peptide (GIP) [11–14]. Consistent with this, mice lacking Gα-gustducin or T1R3 demonstrated deficient incretin production and glucose tolerance after administration of glucose in the gastrointestinal tract [18–20].

In cultures of pancreatic islets or the glucose-responsive β-cell line MIN6, T1R-related mechanisms of glucose regulation involve insulin secretion [16, 17, 21]. However, physiological importance of pancreatic sweet taste receptors in control of blood glucose level *in vivo* was examined in only a few studies, which did not fully confirm it. In fasted mice, deletion of T1R2 or T1R3 did not affect glucose tolerance after systemic administration of glucose, which bypasses the intestinal lumen and thus does not induce secretion of incretins [19, 22]. This lack of consistency between the *in vitro* and *in vivo* studies may be due to differing nutrition status of cells in these two types of experiments. While *in vitro* studies use cell cultures supplied with nutrients, *in vivo* studies typically involve testing food-deprived mice. Overnight fasting (typically for 16–18 h) provokes in mice, which are nocturnal and eat during nighttime, a catabolic state and substantial reduction of incretins and insulin release, as well as changes in insulin sensitivity [23–25]. In contrast to humans, in rodents prolonged fast also enhances insulin-stimulated glucose utilization [26, 27]. Thus, overnight fasting is considered more useful for studies of glucose utilization (e.g., effects on muscle uptake of glucose), whereas reduced fast duration is better for assessing insulin action within a more physiological context [24].

Therefore, we compared glucose tolerance of nonfasted *Tas1r3* knockout [28] and wild-type mice to examine the *in vivo* importance of the extraoral T1R3 taste receptor protein in controlling blood glucose homeostasis. To assess the role of T1R3 in the effect of incretins, we compared glucose clearance after intragastric or intraperitoneal administration of glucose. Additionally, there is substantial evidence showing that aging is associated with decreased glucose tolerance, primarily due to impairment of β-cell sensitivity to glucose, decreased insulin production, and increased tissue tolerance to insulin (for review see [29, 30]). To examine whether aging could affect involvement of extraoral sweet taste reception in glucose metabolism, we have studied effects of *Tas1r3* deletion on glucose and insulin tolerance in mice of different ages. We confirmed the role of the oral T1R3 receptor in behavioral studies assessing taste responses to sucrose in *Tas1r3* knockout and wild-type mice.

## Materials and Methods

### Animals

The described experimental procedures have been approved by the Institutional Animal Care and Use Committee (IACUC) at the Pavlov Institute of Physiology (Animal Welfare Assurance #A5952-01). The study was performed with 8- to 36-week-old male mice of two strains: C57BL/6ByJ bearing the wild-type *Tas1r3* allele, used as control (*Tas1r3+/+*; Jackson Laboratory, Bar Harbor, ME), and C57BL/6J-*Tas1r3* ^*tm1Rfm*^ lacking the entire T1R3 coding region and devoid of T1R3 protein [28] (*Tas1r3-/-* **;** kindly provided by Dr. R. F. Margolskee, Monell Chemical Senses Center, Philadelphia, PA, USA). Separate groups of mice were used in different tests. (Numbers of mice are shown in the table and figures below.) During the study, animals were housed individually (taste tests) or by 4–5 in standard polycarbonate cages on a 12-h light-dark cycle (lights on at 8:00 a.m.) in a temperature- and humidity-controlled room. Throughout the study, mice were fed with a standard lab chow (PK-120, MEST Ltd., Moscow, Russia) containing 67% carbohydrates, 5% lipids, and 19% proteins, with an energy value of 13,000 kJ/kg; food and tap water were available ad libitum.

### Taste tests

Behavioral taste responses to 0.03–0.93 mol/L sucrose (Sigma-Aldrich St. Louis, MO, USA) were assessed in separate groups of mice using the brief-access licking test (BALT) and the 48-h two-bottle preference test. The BALT was conducted during the light period using procedures similar to those described by Glendinning et al. [31]. Before testing, mice had restricted access to water (1.5 mL for 22–23 h), while access to food remained unlimited. During the test session, an animal was exposed in gustometer Davis MS-160 (DiLog Instruments, Tallahassee, FL, USA) to three repetitive blocks of stimuli, each consisting of eight trials: six concentrations of sucrose presented in ascending order, and two presentations of distilled water as "washout," one before each of the two highest sucrose concentrations. Access to each solution lasted for 5 s, with 20-s interpresentation interval. Licking ratio was calculated as the percentage of licks to sucrose solution relative to the mean number of licks in the two preceding trials with water. In the 48-h two-bottle tests [32], mice in their home cages had free access to two tubes containing distilled water or sucrose solution. Positions of tubes was changed after 24 h. Sucrose preference was calculated as consumed sucrose solution as a percentage of total fluid intake.

### Glucose and insulin tolerance tests

Glucose and insulin tolerance tests were started at 3–4 p.m. (7–8 h after the beginning of the light period). In the glucose tolerance test (GTT), matching specific recommendations of the Mouse Metabolic Phenotyping Center [25], nonfasted conscious animals received glucose (2 g/kg, 0.1 ml per 10 g body weight; Sigma-Aldrich St. Louis, MO, USA) either intraperitoneally (IP) or by intragastric gavage (IG). Aqueous solution was used in the IG GTT; in the IP GTT, glucose was dissolved in saline. In the insulin tolerance test (ITT), nonfasted mice were injected with insulin (2 U/kg, IP; insulin aspart, Novo Nordisk A/S, Bagsvaerd, Denmark). Blood was sampled by tail cut, and two measurements of glucose concentration for each time point were made 0–120 min after the infusion of glucose or insulin using the One Touch Ultra glucometer (LifeScan, Inc., USA). During the GTT, animals were gently held in custom-made restraint tubes, to which they were habituated during the preceding 2 days. During the ITT, mice were left unrestrained in their home cages.

### Statistical analysis

Statistical analysis was performed using Statistica 7.0 software (StatSoft, Tulsa, OK, USA). Data from the behavioral taste tests and glucose clearance in the GTT and ITT were compared with two-way ANOVA. Concentration (for taste tests) and time (for GTT or ITT) were considered as within-subject factors, and strain was considered as a between-subject factor. Post hoc paired comparisons were made with Fisher’s least significant difference (LSD) test. Blood glucose area under the curve (AUC) was calculated using the trapezoidal rule, and differences between AUCs were assessed with one-way ANOVA. Between-strain comparisons of baseline glucose, body weight, and age were performed using the Student’s t-test. To quantify the correlation of physiological parameters within groups, the Pearson product-moment correlation coefficient was used. All data are presented as mean ± SEM; P values less than 0.05 were considered significant.

## Results

Body weight of *Tas1r3-/-* mice was about 6% greater than body weight of *Tas1r3+/+* mice (t-test, p<0.01), and for both strains it increased with age (Table 1). Baseline blood glucose level was similar in both strains. There was no significant relationship between baseline glucose and body weight or age (Table 1).

**Table 1:** Age, body weight, and baseline blood glucose level in nonfasted *Tas1r3 +/+* and *Tas1r3-/-* mice (combined data from all experiments).

		*Tas1r3+/+* (n = 73)	*Tas1r3-/-*(n = 81)	t-Test
**Characteristic**	**Age range (weeks)**	8–36	8–36	NS
	**Baseline glucose (mM)**	8.67±0.02	8.69±0.01	NS
	**Body weight (g)**	27.23±0.05	29.07±0.05	P<0.01
**Pearson product-moment correlation coefficient**	**Age × baseline glucose level**	-0.02, NS	-0.03, NS	
	**Body weight × baseline glucose level**	0.10, NS	0.07, NS	
	**Age × body weight**	0.65, p<0.05	0.66, p<0.05	

**Fig 1:**
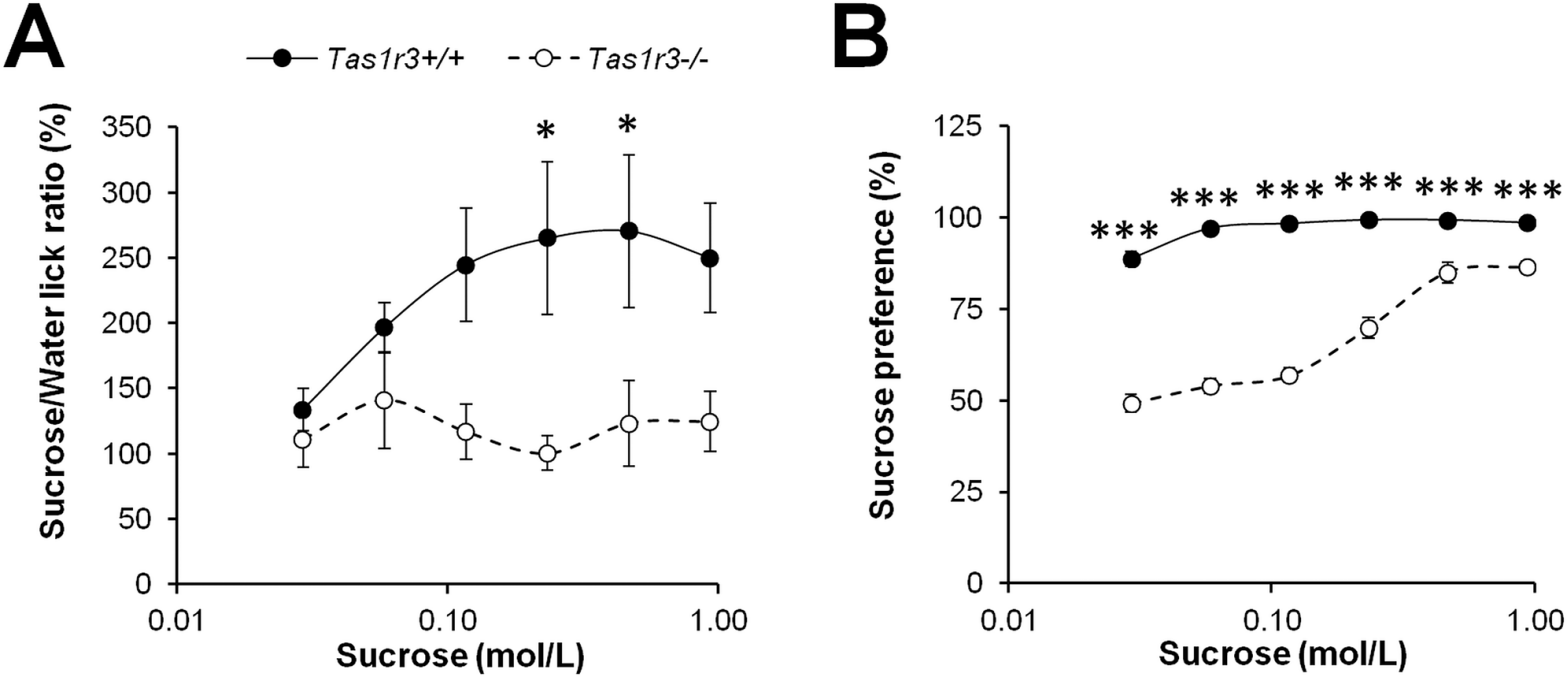
Taste responses to sucrose solutions in naïve *Tas1r3* +/+ and *Tas1r3*-/- mice. A) Licking ratio (%) as a function of sucrose concentration in the brief-access licking test (mean±SEM); n(*Tas1r3*+/+) = 15, n(*Tas1r3*-/-) = 29. B) Sucrose preference scores (%) in the 48-h two bottle test; n(*Tas1r3*+/+) = 18, n(*Tas1r3*-/-) = 12. Post hoc comparisons with Fisher LSD test (*Tas1r3* +/+ vs. *Tas1r3*-/-): *—p<0.05, ***—p<0.001

*Tas1r3-/-* mice had a substantially reduced attraction to sucrose both in the BALT (Fig 1A) and in the 48-h two-bottle test (Fig 1B). Two-way ANOVA of the BALT data revealed significant effects of strain (F(1, 36) = 65.13, P<0.001) and concentration (F(5, 180) = 3.04, p<0.001), as well as strain × concentration interaction (F(5, 180) = 2.33, P<0.01). Mouse strains significantly differed in licking 0.23 and 0.46 mol/L sucrose solutions. A concentration-dependent increase in the licking ratio of sucrose to water was detected for *Tas1r3+/+* mice at concentrations greater than 0.06 mol/L (P<0.01, Fisher LSD test); *Tas1r3-/-* mice licked all concentrations of sucrose at the same rate as they licked water. Similarly, in the 48-h two-bottle tests, two-way ANOVA also revealed strong strain differences in preference for 0.03–0.93 mol/L sucrose solutions (effect of strain: F(1, 28) = 640.42, P<0.0001; effect of concentration: F(5, 140) = 93.44, P<0.0001, strain × concentration interaction: F(5, 140) = 49.68, P<0.0001). Strain comparisons between preference scores at different concentrations confirmed concentration dependence of response. *Tas1r3+/+* mice clearly preferred sucrose to water at concentrations of 0.03 mol/L and higher and showed maximal level of sucrose preference starting at 0.06 mol/L. Knockout mice were indifferent to 0.03–0.12 mol/L sucrose and preferred 0.23 mol/L and higher concentrations (P<0.001, Fisher LSD test).

**Fig 2:**
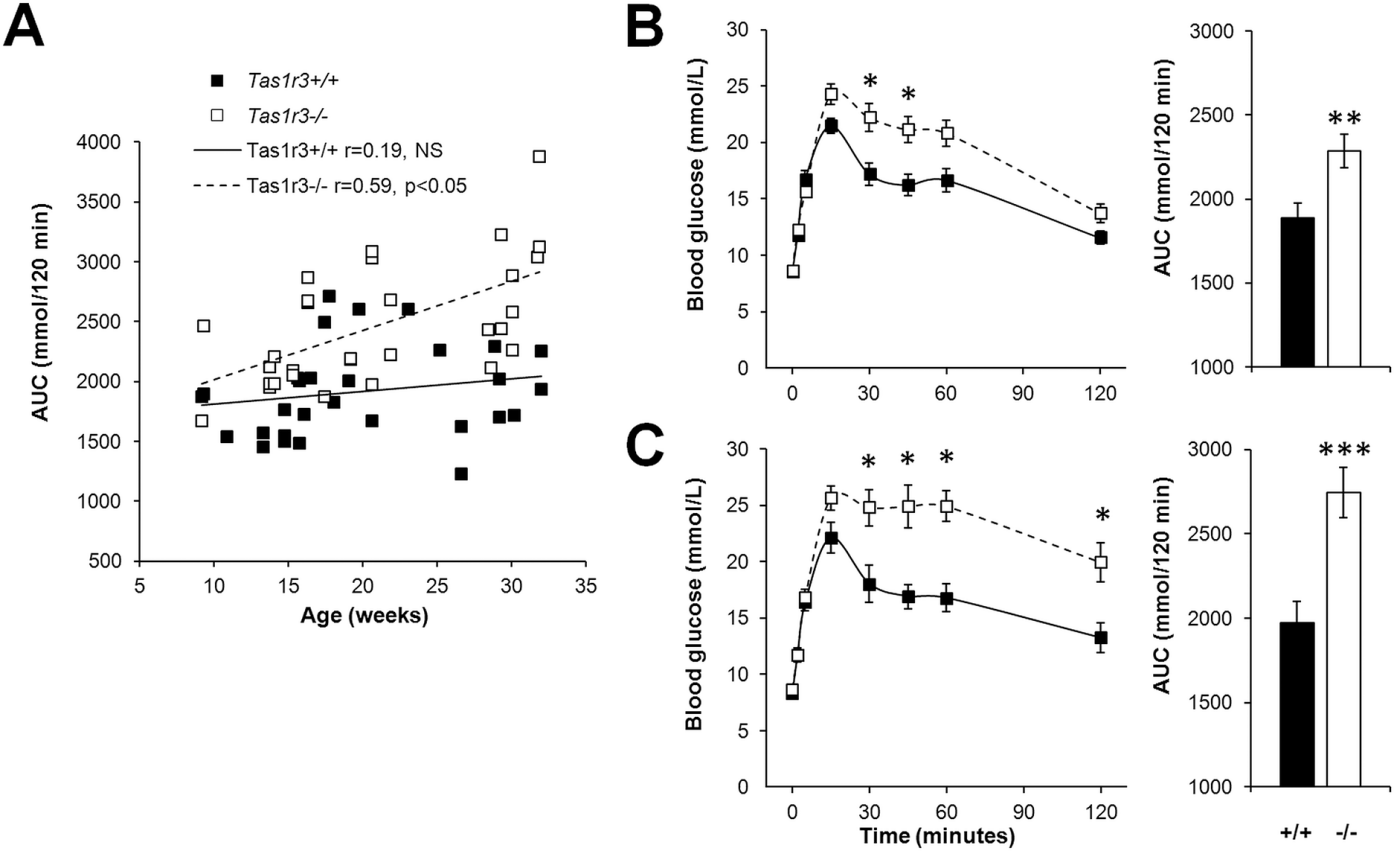
Blood glucose concentration during the intraperitoneal glucose tolerance test (glucose 2 g/kg) in nonfasted *Tas1r3+/+* and *Tas1r3-/-* mice. A) Relationship between glucose AUC and age. Pearson’s coefficient of correlation was calculated; n(*Tas1r3*+/+) = 29, n(*Tas1r3*-/-) = 30. B, C) Blood glucose concentration (left) and glucose AUC (right) in 9- to 21-week-old (B) and 22- to 34-week-old (C) mice. B) n(*Tas1r3*+/+) = 19, n(*Tas1r3*-/-) = 18; C) n(*Tas1r3*+/+) = 10; n(*Tas1r3*-/-) = 12. Post hoc comparisons with Fisher LSD test (*Tas1r3* +/+ vs. *Tas1r3*-/-): *—p<0.05, **—p<0.01, ***—p<0.001.

After IP load with glucose, a significant Pearson’s correlation between AUC of the time course of blood glucose concentration and age was found for *Tas1r3-/-* mice (r = 0.59, P<0.05), while *Tas1r3+/+* mice demonstrated only a nonsignificant tendency of age dependence (Fig 2A). Based on this result, we divided animals of each strain into two age-matched groups (9–21 and 22–34 weeks old) and analyzed within-group differences. *Tas1r3-/-* mice of both ages had significantly impaired glucose tolerance compared with *Tas1r3+/+* mice (Fig 2B and 2C). Although initial peaks of glucose concentrations (15 min after IP administration of glucose) were similar in *Tas1r3-/-* and *Tas1r3+/+* mice, the subsequent decrease of blood glucose level was much slower in *Tas1r3-/-* mice, particularly in the older group. For 9- to 21-week-old mice, two-way ANOVA revealed significant effects of strain (F(1, 35) = 8.80, P<0.01), time (F(7, 245) = 112.11, P<0.000001), and strain × time interaction (F(7, 245) = 6.72, P<0.000001); for 22- to 34-week-old animals two-way ANOVA revealed significant effects of strain (F(1, 20) = 12.60, P<0.01), time (F(7, 170) = 59.95, P<0.000001), and strain × time interaction (F(7, 140) = 6.95, p<0.000001). In the 9- to 21-week-old group, blood glucose AUC of *Tas1r3-/-* mice was about 25% greater than in age-matched *Tas1r3+/+* mice (Fig 2B; F(1, 35) = 9.38, P<0.01, one-way ANOVA); in the 22- to 34-week-old group it was about 75% greater (Fig 2C; F(1, 20) = 15.00, P<0.001, one-way ANOVA).

**Fig 3:**
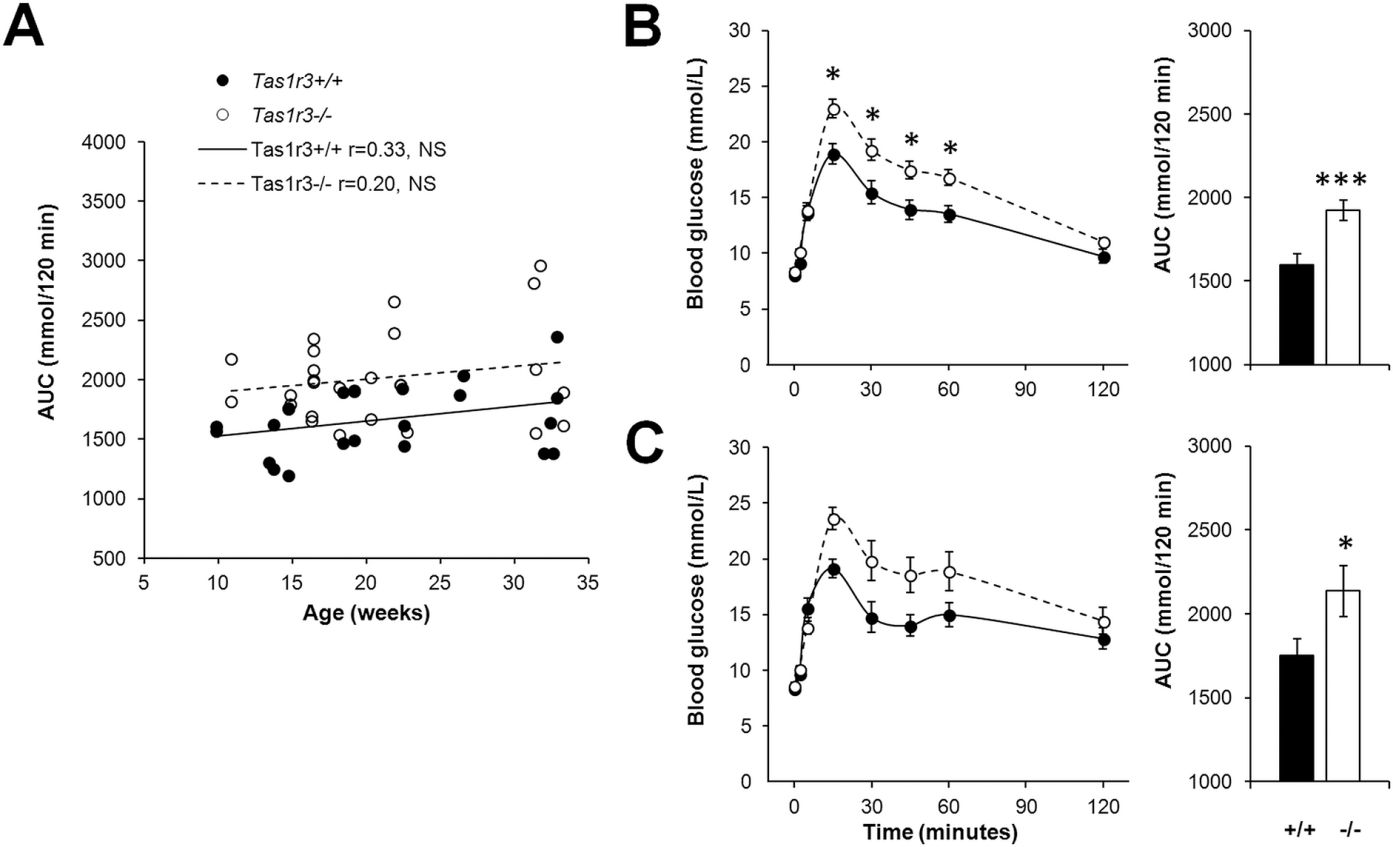
Blood glucose concentration during the intragastric glucose tolerance test (glucose 2 g/kg) in nonfasted *Tas1r3+/+* and *Tas1r3-/-* mice. A) Relationship between glucose AUC and age. Pearson’s coefficient of correlation was calculated. n(*Tas1r3+/+*) = 23, n(*Tas1r3*-/-) = 26. B, C) Blood glucose concentration (left) and glucose AUC (right) in 9- to 21-week-old (B) and 22- to 34-week-old (C) mice. B) n(*Tas1r3+/+*) = 13, n(*Tas1r3*-/-) = 15; C) n(*Tas1r3+/+*) = 10; n(*Tas1r3*-/-) = 11. Post hoc comparisons with Fisher LSD test (*Tas1r3* +/+ vs. *Tas1r3*-/-): *—p<0.05, ***—p<0.001.

In the IG GTT, no significant correlations between blood glucose concentration and age were found for either *Tas1r3+/+* or *Tas1r3-/-* animals (Fig 3A). There was a marked augmentation of blood glucose levels in both age groups of knockout mice compared with *Tas1r3+/+* mice (Fig 3B and 3C). For 9- to 21-week-old and 22- to 34-week-old mice, respectively, two-way ANOVA showed significant effects of strain (F(1, 26) = 16.20, P<0.001; and F(1, 19) = 4.71, p<0.05), time (F(7, 182) = 103.10, P<0.000001; and F(7, 133) = 47.69, p<0.000001), and their interaction (F(7, 182) = 3.76, P<0.001; and F(7, 133) = 4.42, P<0.001). In both age groups, blood glucose AUC was about 30% greater in *Tas1r3-/-* mice than in age-matched *Tas1r3+/+* mice, as confirmed by one-way ANOVA (for 9- to 21- and 22- to 34-week-old mice, respectively: F(1, 26) = 13.52, P<0.01; and F(1, 19) = 4.26, p<0.05; Fig 3B and 3C).

Additional analysis of the data showed an impact of the route of glucose administration, which was similar in both strains (S1 Fig). In general, blood glucose utilization occurred faster after IG infusion than after IP load in mice of both age groups. For 9- to 21- and 22- to 34-week-old *Tas1r3+/+* mice, respectively, two-way ANOVA showed a significant influence of the route of glucose administration (F(1, 19) = 6.40, p<0.05; and F(1, 13) = 15.23, p<0.002), time (F(7, 133) = 35.99, p<0.0001; and F(7, 91) = 40.01, p<0.0001), and their interactions (F(7, 133) = 0.96, p>0.46; and F(7, 91) = 3.60, p<0.002). Glucose AUC was greater in the IP GTT groups than in the IG GTT groups of *Tas1r3+/+* mice (one-way ANOVA: F(1, 19) = 4.88, p<0.05; and F(1, 13) = 12.03, p<0.01, respectively). For 9- to 21- and 22- to 34-week-old *Tas1r3-/-* mice, respectively, two-way ANOVA also showed an effect of the route of glucose administration (F(1, 26) = 5.05, p<0.05; and F(1, 21) = 9.42, p<0.01), time (F(7, 182) = 109.72, p<0.0001; and F(7, 147) = 73.49, p<0.0001), and their interactions (F(7, 182) = 0.94, p>0.47; and F(7, 147) = 2.70, p<0.05). Glucose AUC after IP administration was significantly larger than after IG administration only in the older group of *Tas1r3-/-* mice (one-way ANOVA: F(1, 21) = 8.33, p<0.01) but not in the younger group (F(1, 26) = 3.51, p>0.07).

**Fig 4:**
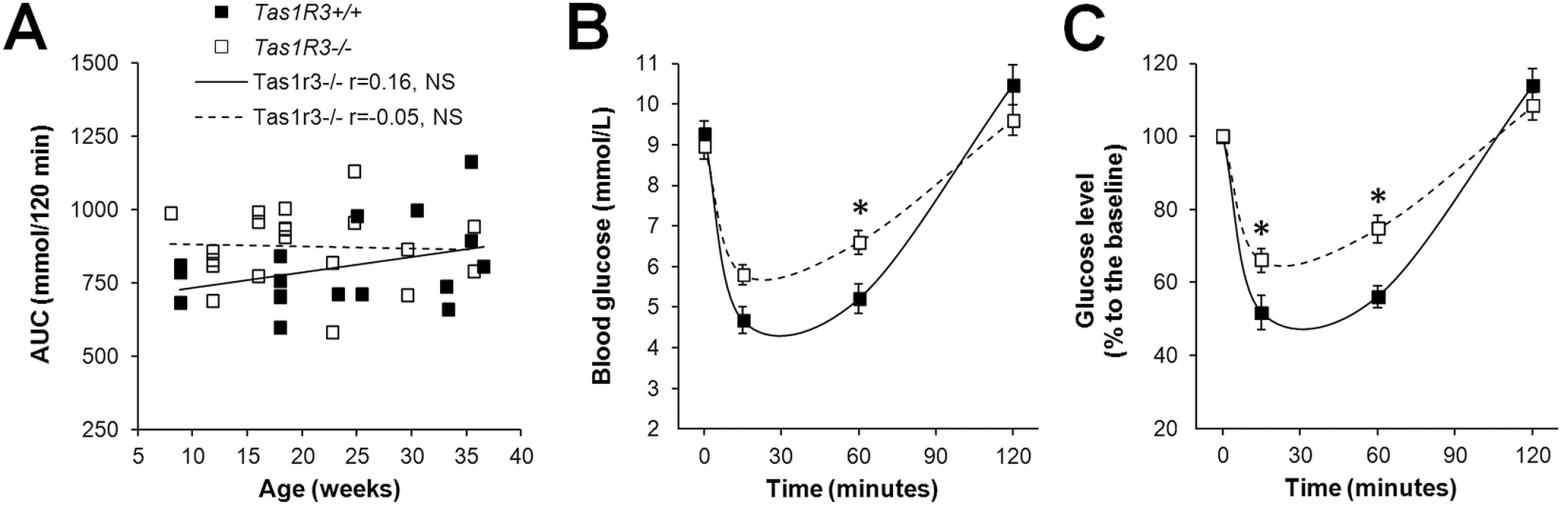
Insulin tolerance test in nonfasted Tas1r3+/+ and Tas1r3-/- mice. A) Relationship between glucose AUC and age. Pearson’s correlation coefficients (r) were calculated. B) Absolute values of blood glucose concentration. C) Percentage relative to baseline level. Insulin (2 U/kg, IP) was injected at zero time point; n(Tas1r3+/+) = 18, n(Tas1r3-/-) = 20. Post hoc comparisons with Fisher LSD test (*Tas1r3 +/+* vs. *Tas1r3-/-*): *—p<0.05

Injection of insulin (2 U/kg, IP) caused a rapid reduction of blood glucose concentration, reaching a minimum 15 min after injection (Fig 4B and 4C). In both strains, basal glucose level was completely restored within 120 min. Hypoglycemia induced by insulin did not depend on age (Fig 4A) or body weight in either strain (data not shown); therefore, calculations were made for the combined group with ages ranging from 8 to 36 weeks. Analysis of both absolute data (Fig 4B) and percentage of basal glucose level (Fig 4C) demonstrated that *Tas1r3*-/- mice had impaired sensitivity to insulin. For absolute values, the two-way ANOVA revealed a significant effect of time (F(3, 108) = 211.16, P<0.000001) and strain × time interaction (F(3, 108) = 11.34, P<0.01); effect of strain was nonsignificant. Fisher’s LSD post hoc test revealed a difference in glucose levels between *Tas1r3*-/- and *Tas1r3*+/+ mice at 60 min (Fig 4B; P<0.05, n = 20–31). Comparisons of values normalized relative to baseline showed significant effects of strain (F(1, 36) = 4.79, P<0.05), time (F(2, 72) = 167.10, P<0.000001), and strain × time interaction (F(2, 72) = 8.63, P<0.001). Post hoc tests confirmed difference in normalized glucose levels between *Tas1r3*-/- and *Tas1r3*+/+ mice at 15 and 60 min (Fig 4C; Fisher’s LSD, P<0.05).

## Discussion

Our results support the important role of cell membrane sensing of sweeteners with T1R3 taste receptor protein at different levels of control of carbohydrate ingestion and homeostasis. Mice lacking the *Tas1r3* gene demonstrated reduced behavioral taste responses to sucrose: they had totally suppressed attraction to sucrose when they were presented with different concentrations for 5-s periods in the BALT (Fig 1A) and had lower preferences for sucrose than *Tas1r3+/+* mice in the 48-h two-bottle test. However, unlike during the BALT, during the long-term preference tests *Tas1r3-/-* mice preferred higher concentrations of sucrose (≥0.23 mol/L) over water (Fig 1B). These data are consistent with earlier studies of *Tas1r3-/-* mice, which revealed a residual behavioral preference of caloric sugars but not nonnutritive sweeteners such as sucralose, acesulfame K, SC-45647, and saccharin, in long-term preference tests [28, 33]. Both *Tas1r3*-independent pre- and postingestive mechanisms could be involved in the observed residual preference of sucrose. The *Tas1r3*-independent preingestive effects are supported by results from a recent study with *Tas1r3-/-*, *TRPM5-/-*, and Gα-gustducin-knockout mice, in which chorda tympani nerve activity in response to sucrose was reduced by roughly 80% relative to wild-type mice but responses to glucose were reduced only by 40% [34]. This suggests that in taste cells, sugars such as sucrose and glucose can activate an additional taste transduction pathway that does not require T1R3, gustducin’s Gα subunit, or TRPM5 and was proposed to be metabolic and K_ATP_ channel dependent [35]. The *Tas1r3*-independent postingestive factors likely include conditioned flavor preference reinforced by nutritive value of sucrose [36].

Our results demonstrate that in nonfasted mice *Tas1r3* deficiency markedly worsens glucose tolerance, regardless of whether the route of glucose administration is intragastric or intraperitoneal (Figs 2 and 3), indicating possible involvement of T1R3-mediated glucose sensing in intestinal enteroendocrine, pancreatic, and/or brain mechanisms controlling glucose metabolism. It is well established that T1R3 is expressed in a variety of tissues beyond the tongue and gut mucosa (e.g., 9–15); however, it is still not clear to what extent these extraoral taste receptors are involved in control of carbohydrate metabolism. In early studies in the human pancreas, T1R3 was immunolabeled in excretory ducts and centroacinar cells, but the endocrine portion of the gland was immunonegative [37]. Later, RT-PCR showed expression of the *TAS1R3* gene in human pancreatic islets [22] and in MIN6 cells, a glucose-responsive β-cell line [16]. Mouse islets [2] and MIN6 cells [17] express elements of intracellular taste signal transduction cascade as well. The sweet taste receptor system of mouse pancreatic β-cells and MIN6 cells seems functional since artificial sweeteners are able to stimulate insulin secretion, which was attenuated by gurmarin, an inhibitor of the mouse sweet taste receptor [16, 22]. In human pancreatic islets, potentiation of insulin release induced by fructose was suppressed by lactisole, an allosteric inhibitor of human T1R3. Further, *in vitro*, genetic ablation of T1R2 or T1R3 led to substantial reduction of the effect of sweeteners on insulin output from mouse islets [19, 22].

In contrast with these results of *in vitro* studies, recent *in vivo* studies in food-deprived mice revealed that the lack of T1R2 [22] or T1R3 [19] had no significant effect on the blood glucose level after IP administration of glucose, although after IG glucose administration *Tas1r3*-/- mice had higher blood glucose and lower plasma insulin levels than did wild-type controls [19]. A likely explanation for this discrepancy between *in vitro* and *in vivo* results is the difference in nutrition status of cells. In cultured mouse islets, positive effects of fructose or noncaloric sweeteners on insulin secretion require presence of an optimal glucose level in the medium. For instance, a sharp reduction of glucose concentration in islet media abolished the potentiating effect of fructose [22] and stimulated activity of noncaloric sweeteners [16] in MIN6 cells. Therefore, pre-experimental fasting can also influence results of *in vivo* experiments. Overnight fasting provokes a catabolic state in mice, which have a unique metabolic response to prolonged fasting that differs from the response to fasting seen in humans. Specifically, fasting impairs insulin-stimulated glucose utilization in humans but enhances it in normal mice [26, 27]. In mice and rats, fasting, or even mild caloric deprivation, leads to the increase in insulin binding in the tissues [38, 39]. Earlier, we found out that effect of T1R3 ablation on glucose utilization was more pronounced in euglycaemic state than after fasting [40]. The present data show that in mice in the nonfasted state, when β-cells are already partially depolarized due to K_ATP_-dependent mechanisms [22, 35] and maintain basal levels of insulin secretion, deletion of T1R3 causes a significant impairment of glucose tolerance in both IP GTT and IG GTT. Thus, the apparent discrepancy between our data and these previous results is likely due to prolonged fasting used in these other studies, which likely caused marked changes in blood glucose regulation mechanisms.

Potentially, T1R3-signalling could be also involved in regulation of glycogenolysis and/or gluconeogenesis. In the adipose tissue, T1R3-signalling induced by non-caloric sweeteners stimulates adipogenesis and suppresses lipolysis [41]. T1R3 is expressed in excretory ducts of the liver [37], where it probably does not interact with glycogenolysis. However, an involvement of T1R3-dependent mechanisms in fat, liver and other tissues in direct or indirect control of glycogen breakdown and gluconeogenesis in our study is unlikely because animals in all our experiments were in non-fasting state, in which synthesis of glucose from polysaccharides and non-carbohydrates is suppressed.

Additionally, our results demonstrate that in the IP (but not IG) GTT, the effect of T1R3 deletion was age related (Fig 2A), suggesting that normal T1R3-mediated extraoral sensing of sweeteners somewhat prevents deterioration of glucose tolerance with age. Decreased insulin secretion due to the loss of β-cell mass or impaired β-cell function and increased insulin resistance are considered two major factors leading to impaired glucose tolerance in the elderly [42–44].

According to the classical concept, the oral ingestion of glucose stimulates more insulin release than does intravenous infusion while causing a similar elevation of the plasma glucose level [45]. This phenomenon, known as the incretin effect, is largely attributable to two insulinotropic hormones released in response to food ingestion from intestinal enteroendocrine K-cells (GIP) or L-cells (GLP-1). Both GIP and GLP-1 have direct stimulatory effects on pancreatic β-cells (for review see [46]). The combined action of incretins is believed to account for about 50% of the total insulin secretory response after a meal [47]. In recent years, sweet taste molecules, including T1R3, as well as intracellular taste signal transduction machinery in the gut enteroendocrine cells were described among the regulators of incretin production. Immunolabeling has revealed taste signal transducing elements in a number of intestinal L-cells, ranging from 15% in mouse jejunum up to 90% in the human duodenum [11, 48], whereas K-cells likely express only marginal levels of sweet taste protein transcripts [14]. The artificial sweetener sucralose administrated to the mouse enteroendocrine GLUTag cell line or to the human L-cell line NCI-H716 enhances GLP-1 output that could be blocked by species-specific inhibitors of the sweet taste receptors [14, 18]. Knockout mice lacking T1R3, or ileum explants from these mice, showed markedly reduced GLP-1 release in response to luminal infusion of glucose [19, 20]. Consistent with this and with our results (Fig 3), *Tas1r3-/-* mice had higher blood glucose and lower plasma insulin levels during an oral glucose challenge compared with wild-type controls [19]. However, in mouse or rat duodenum and jejunum, only a small number of taste proteins are colocalized with enteroendocrine cells [13, 49], and there is still no convincing evidence that T1R3-dependent intestinal endocrine mechanisms are potent enough to control blood glucose levels *in vivo*.

In our study, like in classical investigations [45], the involvement of T1R3 in regulation of intestinal secretion of incretins could be evident by comparing blood glucose clearance after administration of the same dose of glucose by different routes. We show that both *Tas1r3+/+* and *Tas1r3-/-* mice demonstrated similar incretin effects (S1 Fig): in both types of mice blood glucose clearance was more active after IG glucose administration than after IP administration. Pancreatic β-cells and gut enteroendocrine cells use a common metabolic mechanism of glucose sensing, which requires glucose transporter GLUT2, the glycolytic enzyme glucokinase, and the K_ATP_ channel [50–52]. Therefore, because the route of glucose administration affected blood glucose clearance in *Tas1r3-/-* mice, we suggest that in the euglycemic state K_ATP_-dependent metabolic mechanisms predominantly determine gut regulation of the glucose homeostasis.

Impaired glucose tolerance is usually associated with reduced insulin sensitivity, which was also demonstrated for *Tas1r3-/-* mice in our study (Fig 4A). Higher body mass of *Tas1r3-/-* mice could have contributed to their lower insulin sensitivity, but the difference in body weight was small (about 6%, Table 1), and body weight did not correlate with glucose level. Reduction of insulin tolerance also did not correlate with age (Fig 4B) and body weight. Therefore, higher body weight of Tas1r3-/- mice seems insufficient to explain their reduced insulin sensitivity. Another possible cause of decreased insulin sensitivity of *Tas1r3-/-* mice could be chronic elevation of postprandial glucose level, which was shown in our glucose tolerance experiments. In particular, raised blood glucose levels cause overactivity of the hexosamine biosynthesis pathway of glycolysis via modulation of transcriptional factors by *O-N*-acetylglucosamine, including transcriptional factors of the insulin receptor substrate and probably GLUT4 (for review see [53]), which may lead to reduced insulin sensitivity observed in *Tas1r3-/-* mice.

There is evidence that in addition to the gastrointestinal tract and pancreas, the central nervous system may have sweet taste signaling mechanisms that play an important role in regulating glucose homeostasis and therefore may be involved in effects of T1R3 deficiency found in this study. The fall of central glucose levels causes a sequence of neurohormonal reactions known as feedback response launched mainly by activation of glucose-sensing neurons in ventromedial hypothalamic nuclei, orexin neurons in perifornical area, and neurons in the brainstem [54–56]; this includes sympathoadrenal activation followed by increases of plasma epinephrine, norepinephrine, and glucagon, which in turn leads to hepatic gluconeogenesis and inhibition of pancreatic insulin secretion [57]. An acute increase in central glucose, which likely occurs in our experimental protocol, results in an opposite response: an increase in insulin levels and suppression of hepatic glucose production through reduction of gluconeogenesis and glycogenolysis [58]. Several mechanisms of glucose sensing, which do not require intracellular glucose metabolism or glucokinase/K_ATP_ pathways, have been demonstrated in the hypothalamus (for review see [59]). It is quite plausible that glucosensing neurons could use a sweet taste receptor. Ren et al. [15] have reported that T1Rs and α-gustducin are highly expressed in neurons of mouse hypothalamus compared with cortex and hippocampus. Strong expression of T1R2 and T1R3 was found in arcuate and paraventricular nuclei of the hypothalamus, as well as in the medial habenula and the epithelial cells of the choroid plexus. Importantly, the arcuate nucleus is a key region detecting peripheral metabolic status and then relaying this information to other hypothalamic nuclei, including the ventromedial nucleus and paraventricular nuclei 53].

Most of glucose-sensing neurons are glucose inhibited (GI) and reduce their activity during elevation of blood glucose above the euglycemic level. Both glucose-excited (GE) and GI neurons of the hypothalamus are extremely sensitive to glucose changes when extracellular concentrations are less than 2 mM, which occurs at euglycemic blood levels [60], and they have minimal response when glucose levels in the hypothalamus exceed 2 mM, suggesting that these glucose-sensing neurons primarily sense glucose deficit [5, 61]. An additional smaller population of glucose-sensing units is present in the arcuate nucleus, which include high-GE and high-GI neurons responding to an increase in extracellular glucose from 5 to 20 mM; however, it is still not clear whether these neurons play role in regulating hyperglycemic states. It is interesting that glucose sensing of the high-GE and high-GI neurons is K_ATP_ independent [6]. Collectively, there is evidence that glucose-metabolism-independent pathways in the central nervous system may involve T1Rs and take place under control of peripheral glucose homeostasis in the hyperglycemic state; however, their role needs to be elucidated. Additional investigations of neurotransmitter and hormonal specificity of hypothalamic neurons expressing T1R3 possibly will shed light on their physiological relevance.

Measures of glucose metabolism used in this study may have been affected by direct effects of absence of the T1R3 protein in extraoral cells, as well as by indirect effects from these cells to other tissues (e.g., mediated by metabolic, hormonal or paracrine effects). The goal of our ongoing studies is to find out, which of these mechanisms are involved in impaired glucose metabolism in *Tas1r3-/-* mice. If genetic variants of the *Tas1r3* gene alter glucose metabolism in mice, then similar relationships may also exist in humans. Human T1R genes are polymorphic [62], and some of these polymorphisms are associated with taste functions [63–67]. Our study suggests that these functional polymorphisms of human T1R genes may also be associated with glucose metabolism and related diseases in humans. This emphasizes importance of human studies of T1R genes as potential new targets for diagnostics, prevention and treatment of metabolic diseases.

In conclusion, we have shown that the lack of attraction to sucrose demonstrated in *Tas1r3*-/- mice, compared with *Tas1r3*+/+ mice, is associated with reduced glucose tolerance in these mice. In nonfasted mice, the deletion of the T1R3 subunit of the sweet taste receptor results in substantial impairment of blood glucose clearance after both intragastric and intraperitoneal glucose administration. This clearly indicates involvement of the extraorally expressed T1R3 protein in control of glucose homeostasis in hyperglycemic states. Deletion of T1R3 had minor impact on the incretin effect, which suggests that intestinally expressed T1R3 protein is less important for regulation of blood glucose level compared with other extraoral sites, such as pancreas or brain. Reduced glucose tolerance after T1R3 deletion was associated with impaired insulin sensitivity. We have also demonstrated a marked age dependence of the effect of T1R3 receptor protein on blood glucose levels in the intraperitoneal glucose tolerance test. Altogether, our results suggest that further investigation of visceral reception of sugars with the T1R3 protein may lead to therapeutic approaches in the treatment of carbohydrate homeostasis disorders.

## Supporting Information

S1 Fig Effect of intraperitoneal (IP) versus intragastric (IG) administration of glucose (2 g/kg) on blood glucose concentration (left) and glucose AUC (right). Nonfasted *Tas1r3+/+* (A, B) and *Tas1r3-/-* (C, D) mice 9–21 weeks of age (A, C) and 22–34 weeks of age (B, D). Post hoc comparisons with Fisher LSD test (IP vs. IG): *p<0.05, **p<0.001.
(TIF)
