# Supplementary figures and images for: Impaired Glucose Metabolism in Mice Lacking the Tas1r3 Taste Receptor Gene
Source: PLoS One. 2015 Jun 24;10(6):e0130997. doi: 10.1371/journal.pone.0130997 (PMC4479554; doi:10.1371/journal.pone.0130997)

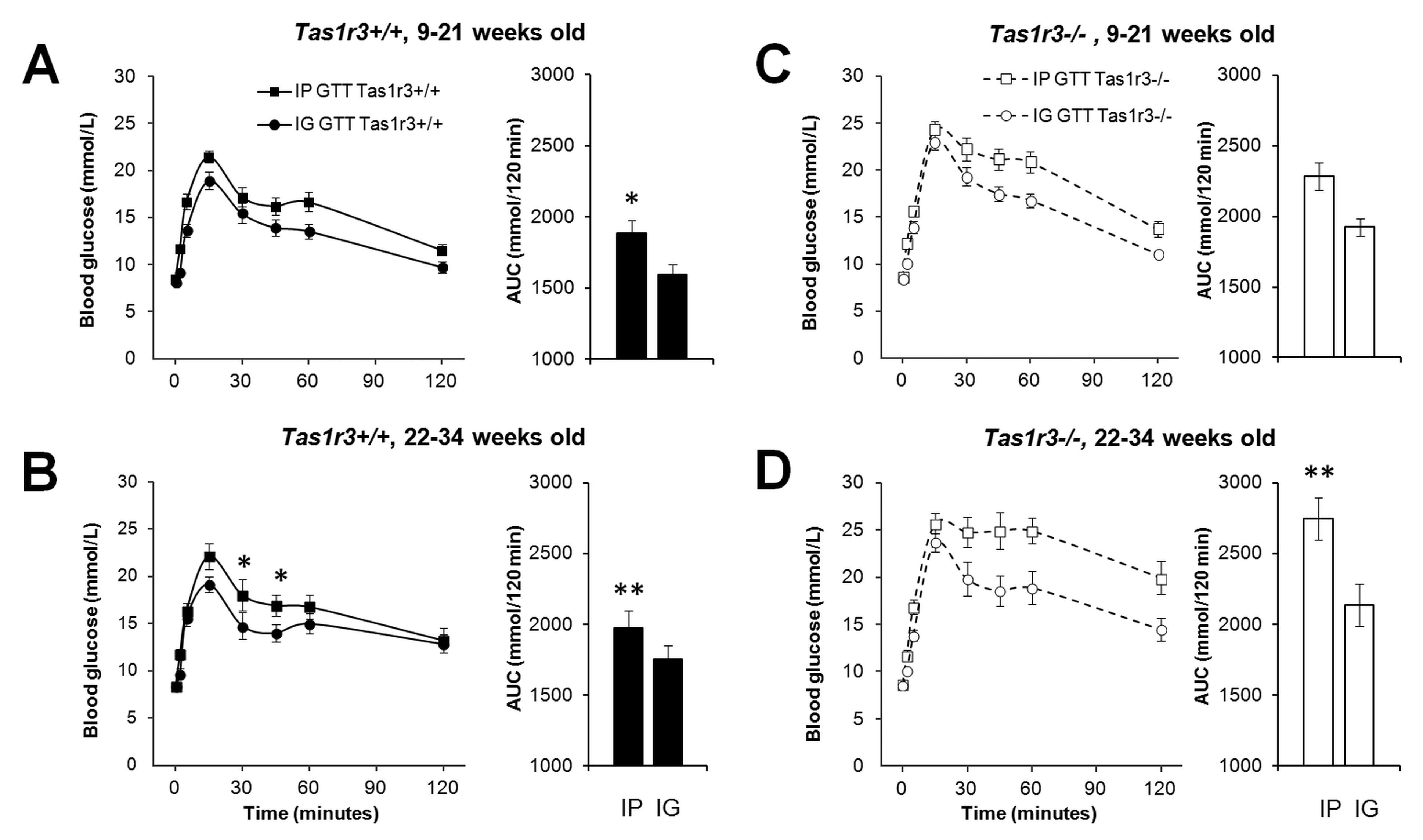

Supplement: S1 Fig — Nonfasted Tas1r3+/+ (A, B) and Tas1r3-/- (C, D) mice 9–21 weeks of age (A, C) and 22–34 weeks of age (B, D). Post hoc comparisons with Fisher LSD test (IP vs. IG): *p<0.05, **p<0.001. (TIF) [file pone.0130997.s001.tif]
